# Supplementary figures and images for: Preserved intention understanding during moral judgments in schizophrenia
Source: PLoS One. 2021 May 19;16(5):e0251180. doi: 10.1371/journal.pone.0251180 (PMC8133419; doi:10.1371/journal.pone.0251180)

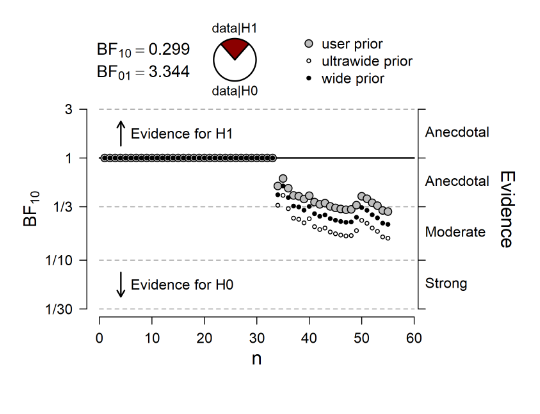

Supplement: S1 Fig — (TIF) [file pone.0251180.s001.tif]

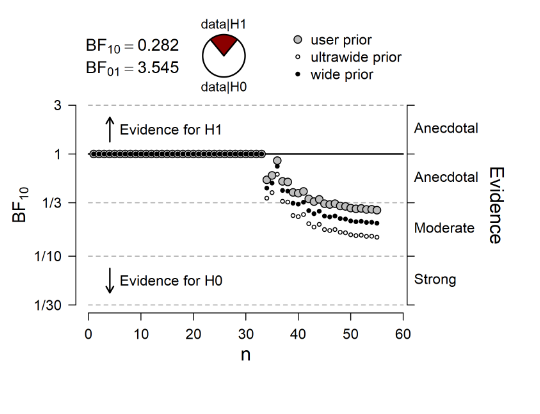

Supplement: S2 Fig — (TIF) [file pone.0251180.s002.tif]

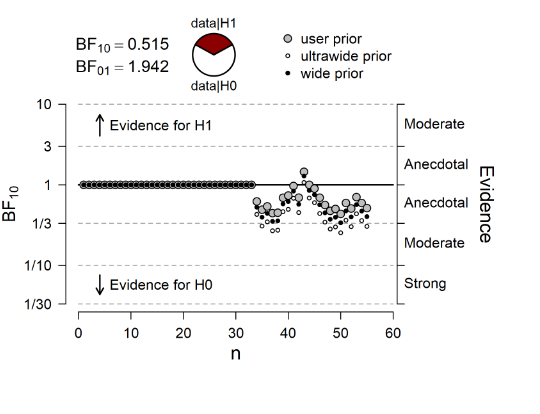

Supplement: S3 Fig — (TIF) [file pone.0251180.s003.tif]

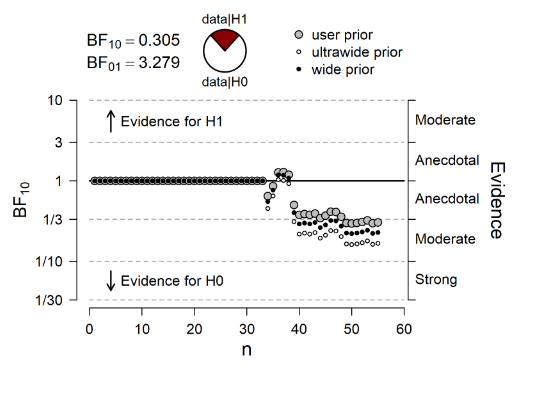

Supplement: S4 Fig — (TIF) [file pone.0251180.s004.tif]
