# Supplementary material for: Preserved intention understanding during moral judgments in schizophrenia
Source: PLoS One. 2021 May 19;16(5):e0251180. doi: 10.1371/journal.pone.0251180 (PMC8133419; doi:10.1371/journal.pone.0251180)

**Preserved Intention Understanding During Moral Judgments in Schizophrenia**

Lisa Kronbichler, Renate Stelzig-Schöler, Melanie Lender, Stefanie Weber, Brandy-Gale Pearce, Luise-Antonia Reich, Wolfgang Aichhorn & Martin Kronbichler

SUPPLEMENTARY MATERIAL

**Methods**

Example Vignettes – English Original

*Accidental harm*.

Grace and her friend are taking a tour of a chemical plant. When Grace goes over to the coffee machine to pour some coffee, Grace’s friend asks for some sugar in hers. There is white powder

in a container by the coffee. The white powder is a very toxic substance left behind by a scientist, and therefore deadly when ingested in any form. The container is labeled “sugar,” so Grace believes that the white powder by the coffee is sugar left out by the kitchen staff. Grace puts the substance in her friend’s coffee. Her friend drinks the coffee and dies.

Putting the substance in was:

1 2 3 4 5 6 7

Forbidden Permissible

*Attempted harm*.

Dan is giving a visitor a tour of a laboratory. Before visitors enter the testing room, all test tubes containing disease antigens must be contained in a chamber by flipping a switch. A repairman has just come to fix the switch, which had been broken. The switch has been successfully repaired, so the test tubes are quite safely contained. Thus, anybody who enters the room will be

safe and unexposed. Dan believes that the switch is still broken after a conversation with the repairman, so he believes it is not safe for the visitor to enter. Dan tells the visitor to enter the testing room. The visitor does not contract any disease and is fine.

Telling the visitor to enter was:

1 2 3 4 5 6 7

Forbidden Permissible

Example Vignettes – German Translation

*Accidental Harm*

Gabi und ihre Freundin besichtigen eine Chemiefabrik. Als Gabi zur Kaffeemaschine geht um Kaffee zu holen, bittet ihre Freundin sie um etwas Zucker in ihren Kaffee. Neben der Kaffeemaschine steht ein Behälter mit weißem Pulver. Dieses Pulver ist ein starkes Gift, welches ein Wissenschaftler hier vergessen hat und ist tödlich, sollte es in irgendeiner Weise in den menschlichen Körper gelangen. Da der Behälter die Aufschrift „Zucker“ trägt, denkt Gabi dass es sich bei dem Pulver um Zucker handelt welchen das Küchenpersonal dort stehen gelassen habe. Sie gibt die Substanz in den Kaffee ihrer Freundin. Ihre Freundin stirbt.

Die Substanz in den Kaffee zu geben war:

1 2 3 4 5 6 7

Moralisch verwerflich Moralisch akzeptabel

*Attempted Harm*

Daniel zeigt einem Besucher ein Labor. Bevor Besucher den Testraum betreten können, müssen alle Reagenzgläser welche Krankheitserreger beinhalten in einer Kammer verschlossen werden. Dazu muss ein Schalter betätigt werden. Da dieser Schalter nicht mehr funktioniert hatte, kam vor kurzem ein Handwerker um ihn zu reparieren. Der Schalter wurde erfolgreich repariert und alle Reagenzgläser sind sicher verwahrt. Daher ist jeder Besucher, der den Raum betritt sicher und wird nicht den Krankheitserregern ausgesetzt. Nach einem Gespräch mit dem Handwerker glaubt Daniel, dass der Schalter noch immer defekt ist und dass es daher nicht sicher ist den Raum mit Besuchern zu betreten. Daniel ermuntert den Besucher den Testraum zu betreten. Der Besucher wird nicht den Krankheitserregern ausgesetzt und ist gesund und wohlauf.

Den Besucher in den Raum zu schicken war:

1 2 3 4 5 6 7

Moralisch verwerflich Moralisch akzeptabel

**Methods**

Permutation Tests

Permutation tests were calculated using the SPSS Exact Tests extension, providing Monte-Carlo p-values for non-parametric Mann-Whitney U tests.

**Results**

**Bayes Analyses**

Sequential analyses of Bayes Independent Samples T-test (as implemented in JASP) were done to explore the robustness of our effects (Group 1 ≠ Group 2) given the number of participants. Default settings were chosen according to [(Wagenmakers et al. 2018)](https://paperpile.com/c/ygUjU4/R69n). T-test are calculated for each condition contrasting patients with SSD against healthy controls.

Accidental Harm


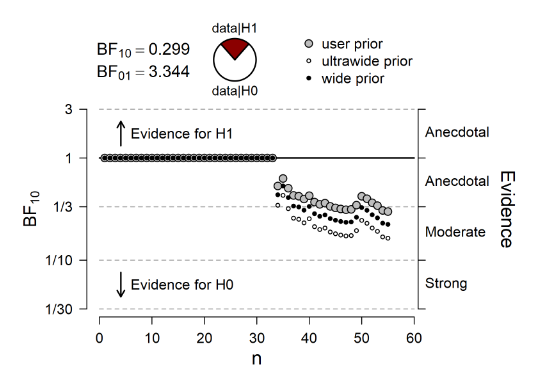


Attempted Harm


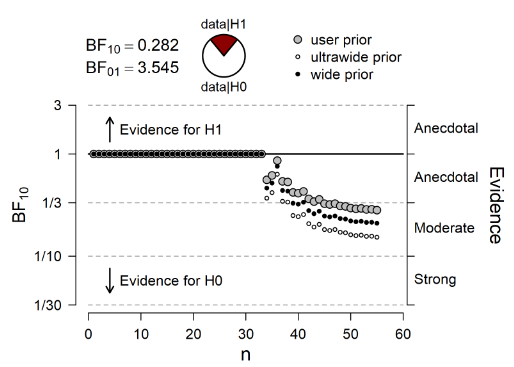


Intentional Harm


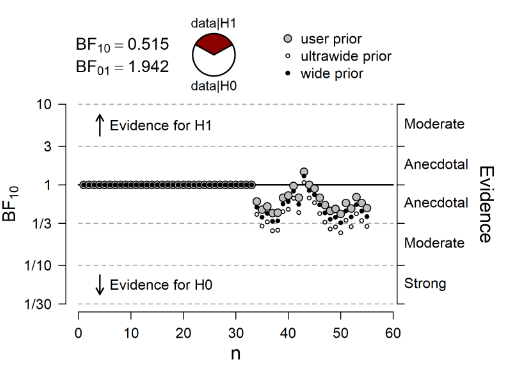


Neutral Acts


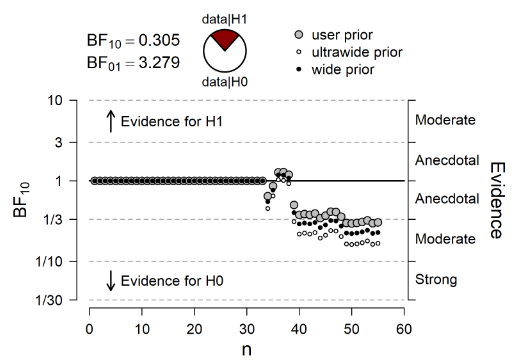


**Permutation Test**


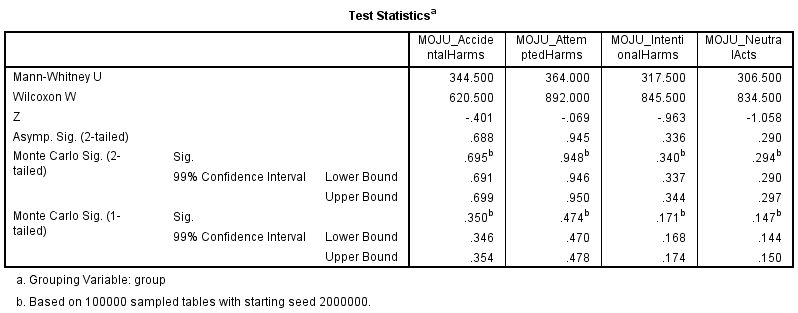

Supplement: S1 File — (DOCX) [file pone.0251180.s005.docx]
